# Lipid signaling in plants

**DOI:** 10.3389/fpls.2013.00216

**Published:** 2013-06-27

**Authors:** Xuemin Wang, Kent D. Chapman

**Affiliations:** ^1^Department of Biology, University of Missouri-St Louis and Donald Danforth Plant Science Center, St. LouisMO, USA; ^2^Department of Biological Sciences, Center for Plant Lipid Research, University of North TexasDenton, TX, USA

Membrane lipids provide both the structural basis for cell membranes and a rich source of cellular mediators that regulate many aspects of plant development and environmental interactions. Over recent years, lipids as hormones and signaling messengers have gained increasing attention in the plant biology community. This volume collected 20 articles, including both primary research articles and several timely reviews on lipid signaling pathways, from active researchers addressing various fundamental questions in lipid signaling in plants (Ibrahim et al., 2011; Alford et al., 2012; Benning et al., 2012; Berkey et al., 2012; Dave and Graham, 2012; Dieck et al., 2012; Ge et al., 2012; Guo and Wang, 2012; Jung et al., 2012; Koo and Howe, 2012; Lorenc-Kukula et al., 2012; Maatta et al., 2012; Pleskot et al., 2012; Scherer et al., 2012; Stenzel et al., 2012; Strawn et al., 2012; Teaster et al., 2012; Wager and Browse, 2012; Xia et al., 2012; Arisz et al., 2013). One important question addressed is what lipids act as mediators in plants, and several classes of lipids and their related metabolites have been described, including phosphatidic acid, oxylipins, phosphoinositides, sphingolipids, free fatty acids, lysophospholipids, *N*-acylethanolamines, oxidatively modified galactolipids, and others. In addition, advances in mass spectrometry-based analysis, which allow sensitive identification of lipids with structural information, have raised many new questions: How many lipids are there in plants? How do plant lipidomes change in response to growth, developmental, and stress cues? As a result, many lipid mediators remain to be identified, and this volume provides a current, baseline knowledge on lipid signaling molecules and their actions in plants.

Signaling lipids are produced and metabolized by a number of enzymes described in this volume, including phospholipase Ds, phospholipase As, acyl hydrolases, phytosphingosine kinases, diacylglcerol kinases, fatty acid amide hydrolases. Each enzyme class has multiple members, which contribute to the spatial and temporal production of lipid mediators, as well as to the influence of specific molecular species for selected actions. Additional molecular complexity is afforded by the fact that each class of lipid mediators may be produced by different enzymes. Different approaches, such as genetic ablation of specific genes, enzymatic kinetics, lipid profiling, or differential metabolic labeling, have been applied. Deciphering the complexity of lipid molecular signals and their metabolism has been a challenge.

Lipid signaling plays diverse roles in various cellular and physiological processes. The involvement of lipid mediators has been discussed here in plant responses to hormones (e.g., abscisic acid and auxin), abiotic stresses, plant-microbe interactions, and in plant growth and development. Some intriguing aspects of plant lipid mediators are also addressed, such as how lipids might play roles as long distance mobile signals in addition to their localized actions, contributing to processes such as flowering and defense against pathogens. One major challenge has been to elucidate mechanistically how lipid mediators carry out their functions. Recent advances in oxylipins, particularly metabolites in the jasmonate pathway, provide an excellent example of how some key players in the signaling cascade that have been identified and interact directly with target proteins to influence changes in gene transcription. At the same time, these articles on oxylipins emphasize the difficulty of assigning functions to lipid mediators when multiple metabolites within a pathway have biological actions (i.e., OPDA, jasmonate and jasmonyl-leu, and probably others). Identifying lipid-interacting proteins represents an exciting area for future research that will improve our understanding of how different signaling networks in plant cells are integrated. However, translating the milieu of lipid metabolite changes in cells into the mechanisms for regulation of physiological processes in plants will remain a formidable challenge in the coming years.

Elsewhere in the volume, the contribution of lipid head-group differences and their potential for selective actions are suggested. The potential roles of phosphoinositides in nuclear function and in the dynamics of membrane trafficking and cell expansion are discussed. In addition, head-group modifications and their metabolites, like the *myo*-inositol phosphates, appear to play a role in energy homeostasis in plants. Furthermore, biophysical studies have provided information on how PA and its phosphorylated product, diacylglycerol pyrophosphate, interact with proteins and/or cell membranes, suggesting a means for different cellular effects of these two metabolically related classes of signaling lipids.

The publication of this book would not have been possible without the efforts of many people. First and foremost are the authors who responded enthusiastically to the call to contribute to the special volume. And the essential critical comments from the many peer reviewers are gratefully acknowledged which provided valuable feedback to ensure the highest quality, and up-to-date information in the articles.
